# Supplementary material for: Polyphasic Characterization and Genomic Insights into an Aerobic Denitrifying Bacterium, Shewanella zhuhaiensis sp. nov., Isolated from a Tidal Flat Sediment
Source: Microorganisms. 2023 Nov 27;11(12):2870. doi: 10.3390/microorganisms11122870 (PMC10745330; doi:10.3390/microorganisms11122870)
Supplement: Supplementary file 1 [file microorganisms-11-02870-s001.zip › microorganisms-2724419-supplementary.pdf]

# Supplementary Figures and Tables

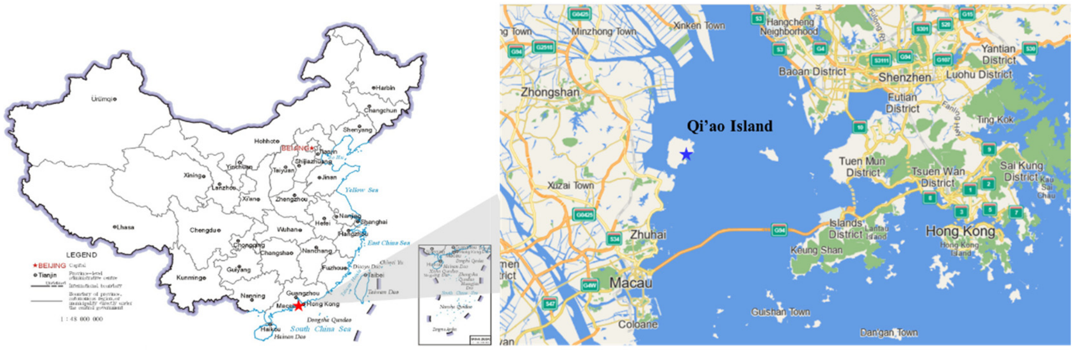


**Figure S1** The sampled site marked by a blue star

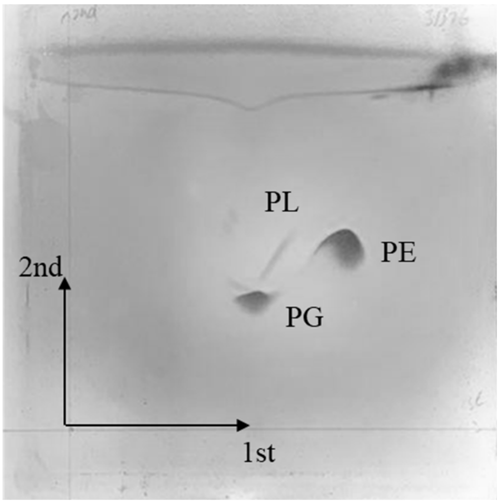


**Figure S2** The polar lipids profile of strain 3B26$^{T}$

Abbreviations: PE, phosphatidylethanolamine; PG, phosphatidylglycerol; PL, phospholipid; 1st, first dimension of TLC; 2nd, second dimension of TLC.

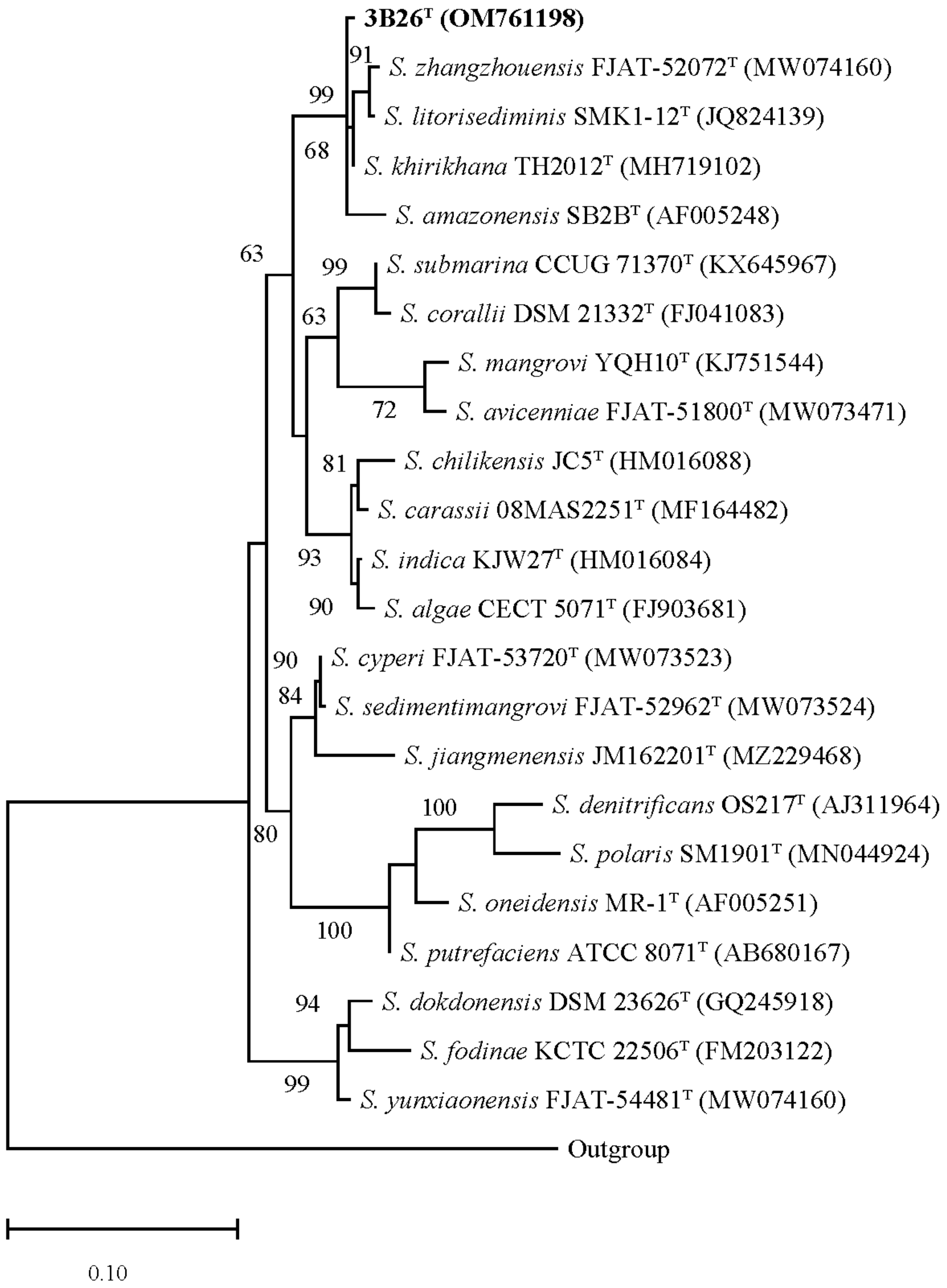


**Figure S3** The maximum likelihood tree based on 16S rRNA gene sequences with a common region including more than 1300 bp shows the phylogenetic relationships between strain 3B26$^T$ and its related reference type strains. Type strain *Alteromonas macleodii* ATCC 27126$^T$ (accession number of its 16S rRNA gene: Y18228) is used as an outgroup. More than 60% bootstrap values are shown at branch points. Bar: 0.1 represents the number of substitutions per site. The accession numbers for the 16S rRNA gene are shown in parentheses.

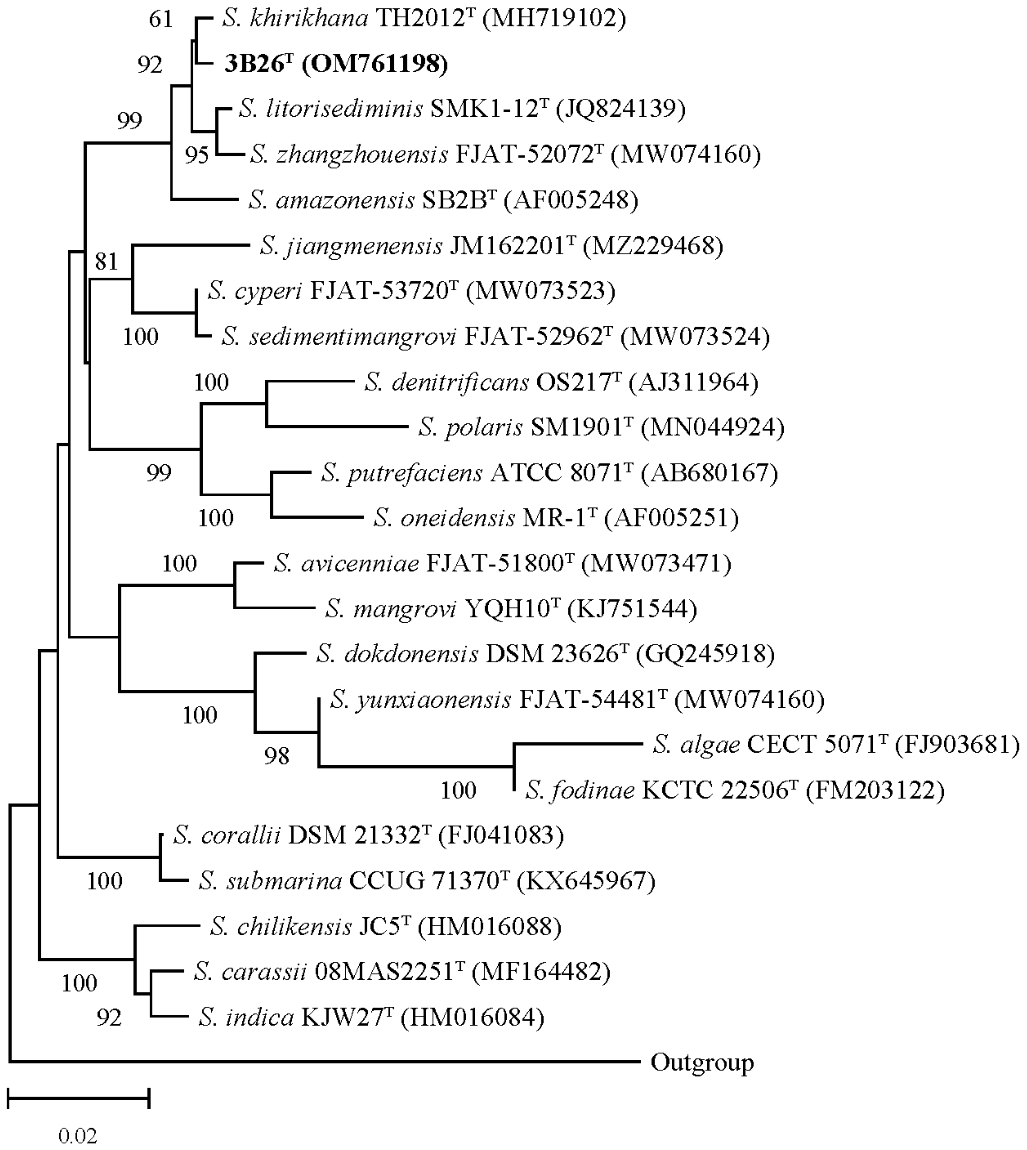


**Figure S4** The minimum evolution tree based on 16S rRNA gene sequences with a common region including more than 1300 bp shows the phylogenetic relationships between strain 3B26$^T$ and its related reference type strains. Type strain *Alteromonas macleodii* ATCC 27126$^T$ (accession number of its 16S rRNA gene: Y18228) is used as an outgroup. More than 60% bootstrap values are shown at branch points. Bar: 0.02 substitutions per nucleotide position. The accession numbers for the 16S rRNA gene are shown in parentheses.

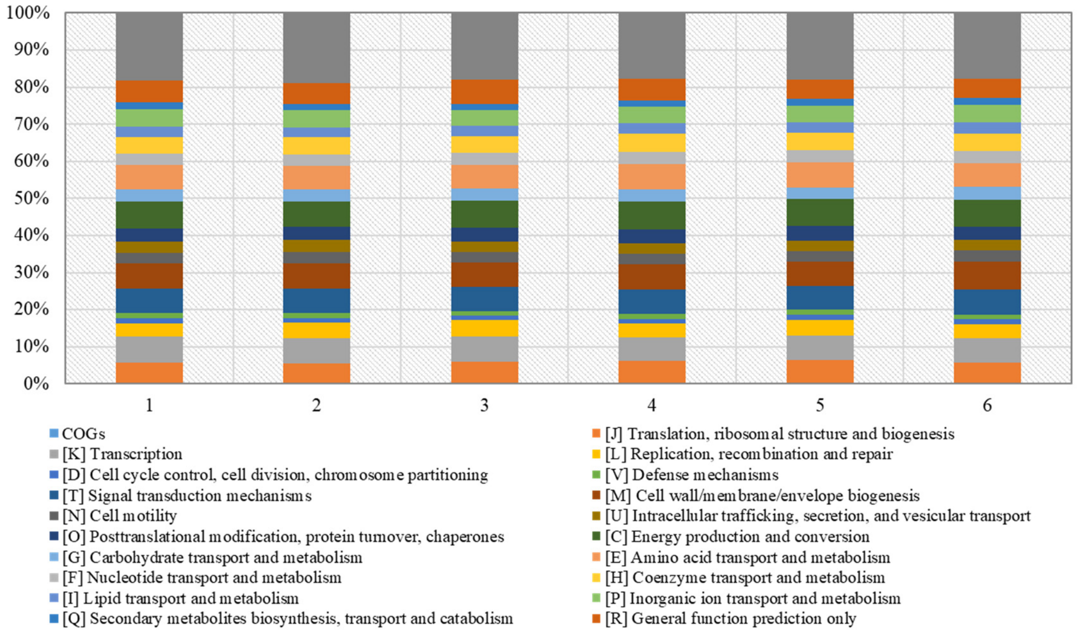


**Figure S5** The distribution of COG categories in each strain

Strains: 1, 3B26$^{T}$; 2, *S. khirikhana* TH2012$^{T}$; 3, *S. zhangzhouensis* FJAT-52072$^{T}$; 4, *S. amazonensis* SB2B$^{T}$; 5, *S. litorisediminis* SMK1-12$^{T}$; 6, *S. jiangmenensis* JM162201$^{T}$.

**Table S1** The general genomic features of strain 3B26$^T$ and reference type strains

| Genomic characteristics | 3B26$^T$ | *S. khirikhana* TH2012$^T$ | *S. zhangzhouensis* FJAT-52072$^T$ | *S. amazonensis* SB2B $^T$ | *S. litorisediminis* SMK1-12 $^T$ | *S. jiangmenensis* JM162201$^T$ |
|---|---|---|---|---|---|---|
| Size (bp) | 4,682,650 | 4,808,629 | 4,513,856 | 4,306,142 | 4,237,767 | 4,436,316 |
| Contig number | 18 | 1 | 1 | 1 | 1 | 33 |
| DNA G + C content (%) | 54.8 | 54.9 | 53.7 | 53.6 | 54.0 | 55.0 |
| Gene number | 4150 | 4300 | 4007 | 3836 | 3817 | 3889 |
| CDS number | 4083 | 4174 | 3850 | 3709 | 3688 | 3788 |
| rRNA gene number | 3 | 25 | 25 | 25 | 25 | 9 |
| tRNA gene number | 64 | 101 | 102 | 102 | 104 | 92 |
| Completeness (%) | 100 | 100 | 100 | 100 | 100 | 100 |
| Contamination (%) | 0.62 | 0 | 0 | 0 | 0 | 0.54 |
| Accession numbers | JAKUDL000000000 | CP020373 | CP080414 | NC_008700 | CP069213 | JAHEPS000000000 |
